# Assessing Derawan Island’s Coral Reefs over Two Decades: A Machine Learning Classification Perspective

**DOI:** 10.3390/s24020466

**Published:** 2024-01-12

**Authors:** Masita Dwi Mandini Manessa, Muhammad Al Fadio Ummam, Anisya Feby Efriana, Jarot Mulyo Semedi, Farida Ayu

**Affiliations:** Department of Geography, Faculty of Mathematics and Natural Sciences, University of Indonesia, Depok 16424, Indonesia; muhammad.al95@ui.ac.id (M.A.F.U.); anisya.feby@ui.ac.id (A.F.E.); jarot.mulyo@ui.ac.id (J.M.S.); farida.ayu81@ui.ac.id (F.A.)

**Keywords:** spatial and temporal distribution, coral reef, Random Forest, Support Vector Machine, Classification and Regression Tree

## Abstract

This study aims to understand the dynamic changes in the coral reef habitats of Derawan Island over two decades (2003, 2011, and 2021) using advanced machine learning classification techniques. The motivation stems from the urgent need for accurate, detailed environmental monitoring to inform conservation strategies, particularly in ecologically sensitive areas like coral reefs. We employed non-parametric machine learning algorithms, including Random Forest (RF), Support Vector Machine (SVM), and Classification and Regression Tree (CART), to assess spatial and temporal changes in coral habitats. Our analysis utilized high-resolution data from Landsat 9, Landsat 7, Sentinel-2, and Multispectral Aerial Photos. The RF algorithm proved to be the most accurate, achieving an accuracy of 71.43% with Landsat 9, 73.68% with Sentinel-2, and 78.28% with Multispectral Aerial Photos. Our findings indicate that the classification accuracy is significantly influenced by the geographic resolution and the quality of the field and satellite/aerial image data. Over the two decades, there was a notable decrease in the coral reef area from 2003 to 2011, with a reduction to 16 hectares, followed by a slight increase in area but with more heterogeneous densities between 2011 and 2021. The study underscores the dynamic nature of coral reef habitats and the efficacy of machine learning in environmental monitoring. The insights gained highlight the importance of advanced analytical methods in guiding conservation efforts and understanding ecological changes over time.

## 1. Introduction

A coral reef is an underwater environment where reef-building corals are present in shallow ocean regions. Coral reefs play a crucial role as ocean ecosystems and serve as a poignant example of the threats posed by climate change. These reefs contribute significantly to Earth’s biodiversity, often being referred to as the “rainforests of the seas” [1,2]. Coral reefs contribute to nutrient recycling, aid in the fixation of carbon and nitrogen, filter water, and supply crucial nitrogen and essential nutrients to support the diverse marine life within the food chain [3,4,5]. Coral reefs offer numerous economic advantages, such as recreational opportunities, tourism, safeguarding coastlines, serving as habitats for commercial fisheries, and preserving marine ecosystems. Corals hold significant value for various reasons, including their practical role in protecting coastlines during storm events and sustaining fisheries vital to many communities [6,7]. Indonesia plays a crucial role in maintaining ecological balance, as it hosts 450 out of the 700 globally recognized coral reef species [8,9]. Derawan Island stands out in Indonesia for having the most extensive expanse and diverse array of coral reefs among its islands.

Upon observing the current conditions of coral reefs on Derawan Island, a study is needed to monitor the conditions of coral reef habitats on Derawan Island over time. Monitoring is carried out using remote sensing technology through the use of multispectral imagery. According to [10], sensing is a science that can be used to obtain information on objects, areas, or phenomena through the analysis of data obtained without making direct contact with the object being studied. Remote sensing data can be used to identify coastal typologies, one of which is the coral reef habitat. Multispectral image data can record the conditions of coral reef habitats because it contains visible light, which has a wavelength between 0.4 μm and 0.7 μm. Visible light can penetrate the water column to a depth of 20 m so that objects at that depth can be recorded [11].

Satellite images that will be used in the research on the spatial distribution of coral reef habitats include Landsat 7 and Sentinel-2. Additionally, in assessing the level of capability of the machine learning classification algorithm for spatial resolution, three spatial resolutions were used: low resolution from Landsat 9 satellite imagery, medium resolution from Sentinel-2 satellite imagery [12], and high resolution from Multispectral Aerial Photography [13]. In the process of monitoring changes in coral reef habitats, a classification stage is needed to identify benthic habitats in the coastal area of Derawan. The classification used is a non-parametric classification algorithm, such as Random Forest (RF), Support Vector Machine (SVM), and Classification and Regression Tree (CART) [14]. This classification algorithm will be used to determine the level of precision and accuracy of the machine learning classification algorithm for satellite images that have low resolution, medium resolution, and high resolution. Through these stages, an algorithm will be selected that has the highest accuracy, which will be used to conduct mapping studies of the spatial distribution of coral reef habitats.

Remote sensing technologies with machine learning algorithms have been widely used to study changes in coral reefs. In previous research, i.e., “*Benthic Habitat Mapping Model and Cross Validation Using Machine Learning Classification Algorithm*” [15], on the development of benthic habitat models using machine learning classification and applying classification models in several research areas, in situ integration data on benthic habitats and WorldView-2 imagery were used to parameterize machine learning algorithms. The results obtained in the previous research show that the RF algorithm is more accurate than other algorithms. Meanwhile, in other research, coral habitat mapping using Sentinel-2 and Landsat 9 imagery were used [12] and multispectral aerial photography was also used for habitat mapping. This was possible because Sentinel-2, Landsat 9, and multispectral aerial photography all have wavelengths between 400 and 700 µm [13].

In our coral reef assessment study, we recognized the potential of deep learning, as evidenced in previous research [16,17,18,19,20,21,22]. However, we opted for RF, SVM, and CART due to several factors specific to our dataset and research objectives. The primary factor was the size and complexity of our dataset, which did not necessitate the use of deep learning techniques. Coral reef mapping typically involves smaller datasets, a condition that is not ideal for deep learning. This is because deep learning methods usually require a large volume of data to function optimally and to mitigate the risk of overfitting. In the context of coral reef environments, where collecting extensive data is often challenging, deploying deep learning models becomes impractical. Our dataset, tailored to the specifics of coral reef mapping, lacked the volume required to leverage deep learning effectively. Consequently, we chose RF, SVM, and CART, as these methods have proven to be effective with smaller datasets, which is often the scenario in coral reef mapping studies. Their ability to produce reliable results with limited data made them more appropriate for our research goals.

This study not only focuses on the ecological and conservation significance of these reefs but also introduces innovative methodologies to monitor and analyze their conditions over an extended period (2003, 2011, and 2021), such as in previous research [23,24] using remote sensing for spatiotemporal monitoring of coastal areas. Our research leverages advanced remote sensing technologies and machine learning algorithms to fill a critical gap in long-term coral reef habitat analysis. While previous studies have demonstrated the effectiveness of these technologies in habitat mapping, they often concentrate on shorter time frames or specific coral types. In contrast, our study provides a comprehensive, long-term analysis of the spatial distribution and changing patterns of coral reef habitats in Derawan Island’s coastal area. A key innovation in our approach is the integration of multispectral image data, which include Landsat 7, Landsat 9, Sentinel-2, and high-resolution Multispectral Aerial Photography. These data, covering visible light wavelengths, allow us to penetrate the water column to a depth of 20 m, capturing detailed images of the coral habitats. We employ non-parametric classification algorithms such as RF, SVM, and CART to analyze the data. These algorithms help determine the precision and accuracy of our classifications across various spatial resolutions. Our methodology stands out in its ability to select the most accurate algorithm for mapping the spatial distribution of coral reef habitats, thus enhancing the reliability of our results.

In our study, we emphasized the importance of clarity and interpretability of results, as these aspects are crucial in our research. The methods we employed, particularly the use of RF, SVM, and CART, were chosen for their ability to provide straightforward insights while being efficient and pragmatic given our computational resources and time constraints. Our primary objective was to delineate the spatial distribution patterns and observe changes in coral reef habitats around Derawan Island, Berau Regency, East Kalimantan, during the significant years of 2003, 2011, and 2021. Moreover, we aimed to evaluate the influence of varying spatial resolutions and determine the effectiveness of different machine learning classification algorithms in accurately mapping these habitats.

While there have been notable advancements in remote sensing technologies and machine learning algorithms for coral reef studies, a comprehensive, long-term analysis, especially in the context of Derawan Island, has been lacking. Prior research, including studies like the ‘Benthic Habitat Mapping Model’ that employed Sentinel-2, Landsat 9, and multispectral aerial photography, have effectively showcased the potential of these technologies in habitat mapping. However, these studies often focused on shorter periods or specific coral types, leaving a gap in long-term, detailed analysis. Our research seeks to fill this gap by conducting a detailed, long-term study spanning the years 2003, 2011, and 2021. This approach is designed to deepen our understanding of the spatial distribution and the evolving patterns of coral reef habitats in the coastal area of Derawan Island. Simultaneously, it assesses the impacts of different spatial resolutions and the efficacy of varied machine learning classification algorithms, thereby providing a nuanced perspective on the temporal dynamics of these vital ecosystems and contributing to more effective conservation strategies.

Indonesia, as the largest archipelagic nation globally, is the custodian of the vibrant and diverse coral reefs of the Derawan Islands, which hold immense ecological and conservation significance. Our ambitious study ventured into modeling the spatial and temporal changes of these coral reef habitats over the specified years, harnessing an innovative blend of remote sensing technologies and machine learning algorithms. The cornerstone of our methodology was the acquisition and preprocessing of multispectral image data from Landsat 7, Landsat 9, Sentinel-2, and Multispectral Aerial Photography. This data was pivotal in penetrating the water column to a depth of 20 m, following rigorous correction processes for atmospheric disturbances. We anchored our methodology on robust non-parametric classification algorithms such as RF, SVM, and CART, recognized for their proficiency in complex environmental studies. The essence of our analysis involved applying these algorithms to the meticulously preprocessed satellite data, engaging in an extensive comparative analysis to establish which algorithm yielded the highest accuracy and precision in classifying and mapping the coral reef habitats. A vital component of our study was the long-term temporal analysis, where these methodologies were applied to data spanning across 2003, 2011, and 2021, thereby revealing the dynamic transformations within these ecosystems. We also explored how different spatial resolutions affected the accuracy of our habitat mappings, a fundamental aspect in grasping the capabilities and limitations of our chosen technologies. Ultimately, our study’s methodology was intricately designed to not only chart the spatial distribution of coral reef habitats but also to decipher their evolution over time and the impact of spatial resolutions, offering profound insights into the dynamics of coral reef ecosystems and establishing a solid groundwork for future conservation strategies.

## 2. Materials and Methods

### 2.1. Study Area

Indonesia, recognized as the largest archipelagic country in the world, is home to over 17,000 islands and a coastline extending over 99,093 km², making it a key region for marine biodiversity, particularly coral reefs [25,26]. These ecosystems, accounting for 450 out of the 700 known coral reef species globally, are not only crucial for ecological balance but also for the economic and tourism sectors [8,9]. The coral reefs of the Derawan Islands are famous, and one of the islands stands out for this notable characteristic.

The Derawan Islands, situated in the Berau Regency of East Kalimantan, Indonesia, consist of several islands, including Derawan, Maratua, and Biduk Biduk. This archipelago is renowned for its stunning natural beauty, particularly Derawan Island, which was recognized as a UNESCO World Heritage site in 2005 [27]. The island’s breathtaking beaches and mesmerizing underwater vistas have captivated diving enthusiasts worldwide, earning it the moniker “Pristine Island” due to its unspoiled natural splendor [28].

**Figure 1:**
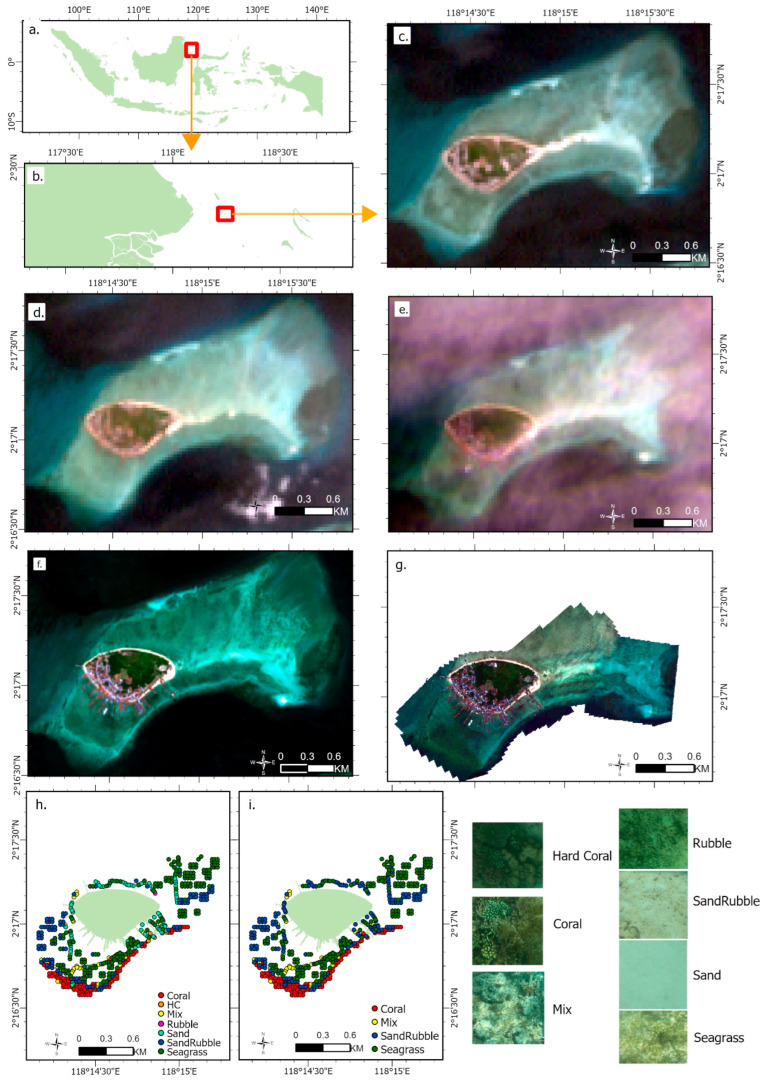
Research Area: (**a**). Indonesia; (**b**). Derawan Archipelago; (**c**). Landsat 7 2003; (**d**). Landsat 7 2011; (**e**). Landsat 9 2022; (**f**). Sentinel-2 2021; (**g**). UAV Multispectral 2021; (**h**). in situ Station in Derawan Island; (**i**) in situ Station in Derawan Island with 4 classification.

The allure of coral reefs stems from their natural beauty. The significance of coral reefs, particularly in regions like Derawan, is multifaceted. Ecologically, they offer habitats for a diverse range of marine life and play a critical role in coastal protection. However, they are increasingly under threat due to environmental changes and anthropogenic activities, such as pollution, overfishing, and climate change, which have led to substantial reef degradation [29,30,31,32,33,34] This alarming trend underscores the necessity for effective monitoring and conservation strategies. Figure 1a illustrates the location of the Derawan Islands within Indonesia, while Figure 1b specifically highlights Derawan Island.

Referring to East Kalimantan Governor Regulation Number 60 of 2019 concerning RZWP3K for the Derawan Islands and Surrounding Waters, Derawan Island is the area with the greatest damage to coral reefs compared to other areas in the Derawan Islands region [35] according to the results of the 2011–2012 Manta Tow survey on the coral around the island. Derawan has HCL cover ranges of 11–25% and 26–50%, and HCL cover of <10% is found mainly on coral reefs near Derawan Island. Based on the data described, it is known that the role of coral reefs is very important in maintaining coastal ecosystems. Based on data from the NOAA Satellite and Information Service, it appears that sea surface temperatures in East Kalimantan in 2002–2003 ranged between 15℃ and 17℃, with bleaching warning conditions in April, June, and November 2002, as well as in April and November 2003. Then, from 2010 to 2011, the sea surface temperature ranged between 15 and 25 degrees Celsius, with a significant change in temperature from 15 degrees in April to 25 degrees in July, which lasted for 10 months, resulting in a level 2 coral bleaching warning; however, in 2011, sea surface temperatures tended to be stable. Furthermore, in 2021–2022, the sea surface temperature ranged between 15℃ and 21℃, whereas in 2020 there was a coral bleaching warning in May–July, with a temperature range of 18℃ to 21℃. Meanwhile, in 2021, sea surface temperature conditions only received coral bleaching warnings in April, June, and October–November [36].

### 2.2. Data

Table 1 presents a comprehensive overview of the datasets utilized in our study, showcasing a range of spatial resolutions and the respective years of data collection, and thereby illustrating the diverse applications of remote sensing technology in our research. It includes data from Landsat 7 and Landsat 9, both offering 30 m resolution imagery from 2003, 2007, and 2022, which underscores the enduring utility of the Landsat program in providing consistent, long-term Earth observations (Figure 1c–e). Additionally, the Sentinel-2 dataset, with a more detailed 10 m resolution, includes data from 2021 and 2022, reflecting our use of the latest satellite imagery available from the European Space Agency for high-resolution analysis (Figure 1f). The table also details Multispectral Aerial Photography data from 2021, featuring a high 8 cm resolution obtained from aerial surveys, essential for our manuscript’s focus on fine-scale, localized studies (Figure 1g). Significantly, the Underwater Photo Transects (UPT) data from 2021, represented on a 50 × 50 cm grid, offer a unique and valuable perspective for our marine environmental assessments, allowing detailed analysis of underwater habitats and ecosystems (Figure 1h,i). Together, these datasets form the foundation of our manuscript’s analytical approach, demonstrating how varying scales and types of remote sensing data can be integrated for comprehensive environmental research.

#### 2.2.1. Landsat-7

Landsat 7 is one of the missions launched by NOAA, NASA, and the USGS. On 15 April 1999, it was launched from Vandenberg Air Force Base in California. The Landsat 7 satellite has an Enhanced Thematic Mapper Plus (ETM+) sensor, which is an advancement of the sensors used by earlier Landsat series. In terms of band composition, Landsat 7 Enhanced Thematic Mapper Plus (ETM+) has eight spectral bands with spatial resolutions of 30 m for Bands 1–7 and 15 m for Band 8 (panchromatic). All bands in Landsat 7 can collect one of two gain settings (high or low) to increase radiometric sensitivity and dynamic range, while Band 6 collects high and low gain for all data [37].

#### 2.2.2. Landsat 9

The Landsat 9 satellite was launched from Vandenberg Air Force Base in California on 27 September 2021, using a United Launch Alliance Atlas V 401 rocket. The Operational Land Imager 2 (OLI-2) sensor was aboard Landsat 9. According to Brian Markham [38], Landsat 9 contains a sensor with 9 bands and a TIRS-2 sensor with 2 bands, comparable to Landsat 9. Band 1 collects ultra-blue waves with wavelengths ranging from 0.435 to 0.451 µm and a resolution of 30 m. Bands 2, 3, and 4 may collect visible waves, particularly blue, green, and red waves, with a resolution of 30 m in the wavelength range 0.452–0.512 µm, 0.533–0.590 µm, and 0.636–0.673 µm, respectively. Band 5 captures NIR waves with a wavelength range of 0.851–0.879 µm and a resolution of 30 m. Bands 6 and 7 may catch SWIR waves with wavelengths ranging from 1.566–1.651 µm to 2.107–2.294 µm at a resolution of 30 m. Band 8 is a band that combines numerous waves to produce a clear black-and-white image; the waves have a wavelength range of 0.503–0.676 µm with a resolution of 15 m. Band 9 is a band that can catch cirrus cloud objects because of its shortwave range, precisely 1.363–1.384 µm, and a resolution of 30 m.

#### 2.2.3. Sentinel-2

The Copernicus program’s Sentinel-2 satellite was launched in June 2015. Sentinel-2 images have spatial resolutions of 10, 20, and 60 m and two types of bands: one that can catch visible and near-infrared (VNIR) waves and one that can record short wave infrared (SWIR). This satellite can employ 12 different types of bands, 9 of which are in the VNIR band and 3 of which are in the SWIR band [39].

#### 2.2.4. Multispectral Aerial Photography

The ability of multispectral aerial photography to sharpen the contrasts in hue between two or more objects is its advantage. Tone sharpening can be employed in multispectral aerial pictures for visual observation without alterations, visual observation with reshoots, and additive color blending with viewing tools [10], With a wave range of 0.4–0.5 µm, the blue channel can capture the reflection of waves that hit the water; hence, it is commonly used for bathymetry, detecting water turbidity, and other water variables. Because the green channel with a wave range of 0.5 µm–0.6 µm can capture the reflection of plant chlorophyll and the amount of chlorophyll in healthy and sick plants differs, the difference can be easily observed by making use of this channel. With a wave range of 0.5–0.6 µm, the Red Channel can be utilized to discriminate between vegetation and non-vegetation objects. The near-infrared (NIR) channel, which has a wave range of 0.7–1.1 µm, can be used to identify the age of vegetation, the location of glacier layers, and the water content of plants. The SWIR channel can be utilized to identify aerosol and water vapor components, mineral types, soil components, and fire potential in dry locations [40].

#### 2.2.5. Underwater Photo Transects (UPT)

The UPT (Underwater Photo Transect) images, numbering a total of 585, captured using GoPro cameras, will undergo a comprehensive analysis on the CoralNet website. CoralNet serves as a dedicated open-source platform specifically tailored for the evaluation of benthic environments. In our study, the categorization of the benthic habitat class will encompass six distinct types: hard coral, soft coral, sand, debris, rock, and seagrass. This extensive collection of UPT images provides a substantial dataset, allowing for a thorough and nuanced analysis of the diverse benthic habitats present in our study area.

### 2.3. Methodology

#### 2.3.1. Data Processing

**Figure 2:**
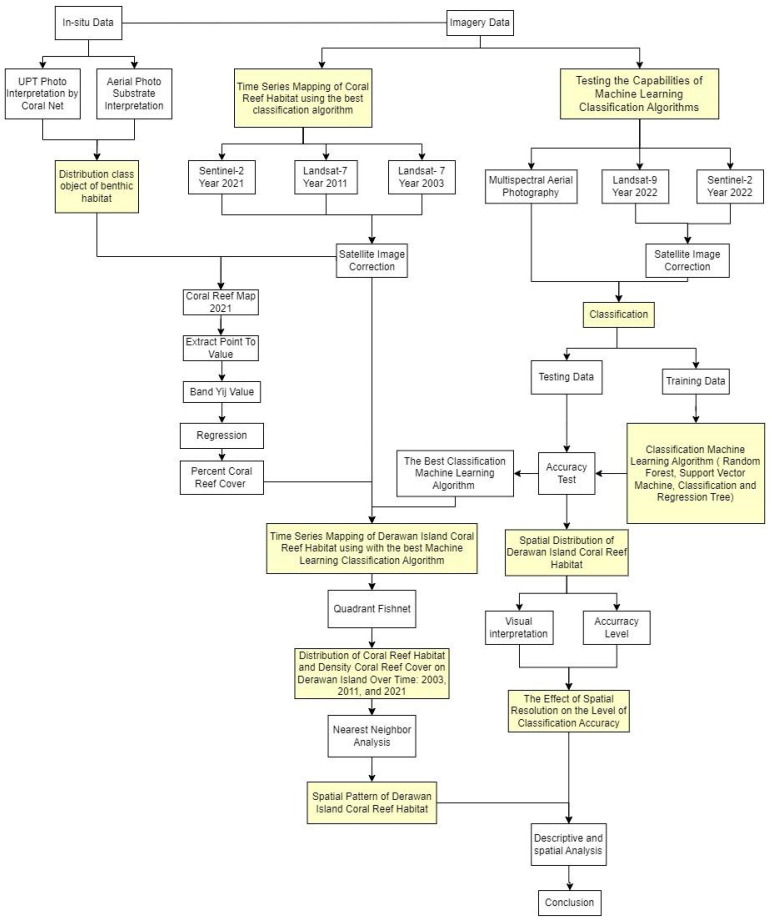
Research Workflow.

**Figure 3:**
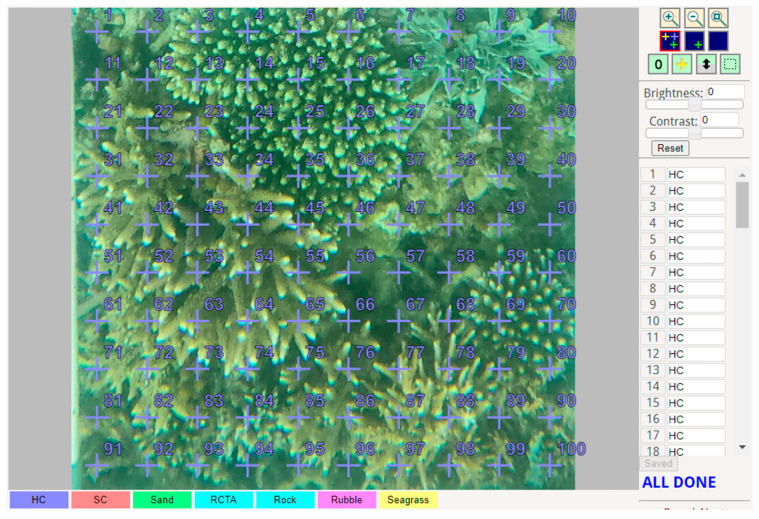
Identify benthic percent cover using CoralNet. The cross mark with numbers of labels shows the point of center grid used to interpret the percent cover of each UPT.

The data processing for this study was divided into two distinct parts. The first part focused on testing the capabilities of various machine learning classification algorithms for accuracy and precision in benthic habitat classification. This was achieved using data from three different sources: Landsat 9 satellite imagery from 2022, Sentinel-2 satellite imagery from 2022, and Multispectral Aerial Photography from 2021. The second part involved time-series mapping of the island’s coral reef habitat. This was conducted using the algorithm that demonstrated the highest accuracy in the previous testing phase and applying it to Landsat 7 satellite imagery from 2003 and 2011 and Sentinel-2 satellite imagery from 2021, as shown in Figure 2 and detailed as follows:

1. **Recapitulation and Validation of UPT Data and Multispectral Aerial Photography**: To calculate the coverage of the coral reef ecosystem, the UPT (Underwater Photo Transect) data was processed using the Coral Net platform [41]. This UPT data, crucial for assessing the ecosystem coverage, involved capturing images with GoPro cameras and GPS sets for precise location data. Each image underwent a detailed analysis using a 10 × 10 grid pattern, totaling 100 points (Figure 3). At this stage, every UPT point represented a 50 × 50 cm area, providing a high-resolution view of the coral reef. This meticulous examination included classifying the benthic environment at each grid point into detailed categories such as hard coral, soft coral, sand, algae, rock, rubble, and seagrass. This comprehensive classification, as depicted in Figure 1h, facilitated an in-depth understanding of the diverse benthic habitats present in each image. Furthermore, the UPT point interpretation of the 50 × 50 cm areas served as a reference to upscale the analysis to larger areas of 10 × 10 m. In this scaling-up process, the classification scheme was simplified into broader categories: coral, sand-rubble, seagrass, and mixed bottom class (Figure 1i). This modification was essential for matching the UPT data with the grid used in Sentinel-2 multispectral aerial photography. This integration of fine-scale UPT analysis and larger-scale satellite data, along with the adjusted classification scheme, allowed for a more comprehensive monitoring and understanding of the coral reef ecosystem. It ensured that the ecosystem was analyzed in a detailed and multi-scaled manner, crucial for accurate ecosystem assessments and conservation efforts.

2. **Benthic Habitat Classification**: Prior to implementing the classification algorithms, we applied necessary corrections to the satellite and UAV imagery, including adjustments for water surface and water column using the Lyzenga algorithm (Depth Invariant Index-Yij) [42]. This preparatory step was crucial to account for variations due to water depth and surface conditions, ensuring a more accurate base for habitat analysis [43,44]. In this phase of our study, we aimed to harmonize the available datasets from different remote sensing platforms. Although we did not have Landsat 9 images from 2021, we used the closest available satellite imagery from 2022, along with Sentinel-2 images from the same year. This was complemented by UAV-based multispectral aerial photos taken in December 2021, coinciding with the date of the Underwater Photo Transects (UPT). This alignment in data collection dates allowed for a coherent framework in our comprehensive benthic habitat assessment, despite the slight temporal mismatch with the Landsat 9 data. Given the spatial resolution limitations of the satellite images, we simplified the detailed UPT classification scheme into broader categories more compatible with the satellite data. These categories included ‘coral’ (encompassing both hard and soft coral), ‘sand/rubble’ (merging sand and rubble), ‘seagrass’, and ‘mixed bottom’ (incorporating algae, rock, and other mixed elements). Such simplification was vital to align with the spatial resolution capabilities of the Landsat 7, Landsat 9, and Sentinel-2 imagery, thereby ensuring a more accurate and feasible classification process. We adapted the ground truth labels for algorithm training to this revised scheme and divided the dataset into training (70%) and testing (30%) sets. The accuracy of each classification algorithm—RF, SVM, and CART—was then assessed using the confusion matrix method, ensuring a robust evaluation of our benthic habitat classification approach.
3. **Temporal Distribution Mapping**: Following the machine learning classification tests, the best-performing algorithm was then applied to a comprehensive timeseries of satellite multispectral imagery. This timeseries spanned from 2003 to 2021 and included data from both Landsat 7 and Sentinel-2 satellites. This approach allowed us to analyze changes in the coral reef ecosystem of Derawan Island over an extended period, providing valuable insights into temporal patterns and trends. To facilitate a balanced and accurate comparison across different years and satellite sources, we resampled the Sentinel-2 satellite image classification results to a 30 m resolution. This was necessary to align with the resolution of the Landsat 7 satellite images. The combined and harmonized dataset was then visualized, where we constructed a detailed map showcasing the temporal distribution and evolution of the coral reef ecosystem from 2003 to 2021. This map not only highlighted the spatial changes but also served as a crucial tool for understanding the long-term environmental dynamics affecting the coral reefs around Derawan Island.
4. **Mapping Changes in Coral Reef Habitat Density**: After the temporal mapping of the coral reef ecosystem around Derawan Island from 2003, 2011, and 2021, we further refined our analysis by focusing exclusively on the coral reef class. We masked out all other classes from our classification results to isolate the coral reef areas. The next critical step involved calculating the density of the selected coral reef class areas, which was derived from our previous classification process. This calculation was achieved through RF regression analysis, correlating the observed coral reef density from the UPT dataset with the Yi band values. These computations utilized image reflectance data from the timeseries and Yij band values from the Depth Invariant Index algorithm. The indicator of coral reef density was an essential component in our study, offering insights into the spatial distribution and density changes of coral reefs over the studied period, thus providing a more nuanced understanding of the ecosystem’s dynamics and health.
5. **Analysis of Spatial Distribution Patterns**: To analyze the spatial distribution and temporal change patterns in the coral reef ecosystem around Derawan Island, we utilized the fishnet tool in GIS. This tool facilitated the creation of a grid-like quadrant area over the entire study region, effectively segmenting the area into smaller, manageable units. This segmentation was crucial for a detailed and systematic analysis of spatial patterns, as it allowed us to examine the distribution of coral reefs within each quadrant and observe variations across different sections of the study area.

#### 2.3.2. Machine Learning Classification

1. Random Forest

Random forest classification is a classification based on a collection of decision trees to significantly increase the accuracy of pattern recognition [45]. RF classification uses a hill-climbing search strategy to find a decision tree that will be the basis for classifying data samples accurately and without errors based on the training data provided [46]. The structure of the RF classification algorithm is divided into several levels of nodes, namely the root node, branch node, and leaf node. Each class is created using a random vector from samples independently, and each decision tree will provide calculations of the most dominant class units to classify certain classes according to the training data [47].

2. Support Vector Machine

Support vector machines (SVMs) were developed by Boser, Guyon, and Vapnik in 1992 [48] during the Annual Workshop on Computational Learning Theory. SVM is a technique for finding a hyperplane that can separate two sets of data from two different classes. SVM is a technique for making predictions in two cases, namely classification and regression. The basic concept of SVM is a combination of computational theories that have existed in previous years. The hyperplane is the best dividing line between the two classes, which can be found by measuring the hyperplane margin and looking for the maximum point [49]. The margin is the distance between the hyperplane and the closest pattern from each class, which is called the support vector. The largest margin can be found by maximizing the distance between the hyperplane and its closest point.

3. Classification and Regression Tree

Classification and Regression Tree (CART) is an algorithmic development of the decision tree technique developed by Leo Breiman, Jerome H. Friedman, Richard A. Olshen, and Charles J. Stone. The development uses a binary recursive partitioning algorithm [50]. CART will produce a classification tree if the response variable has a categorical scale and a regression tree if the response variable is continuous data. The aim of using CART is to carry out classification analysis in the sector of response variables, whether nominal, ordinal, or continuous. The CART method is divided into two methods, namely the classification tree method and the regression tree method. If the dependent variable has a categorical type, CART will produce a classification tree. Meanwhile, if the dependent variable is numeric or continuous, CART will produce a regression tree.

## 3. Result

### 3.1. The Effect of Spatial Resolution on the Level of Classification Accuracy of the Machine Learning Algorithm

**Figure 4:**
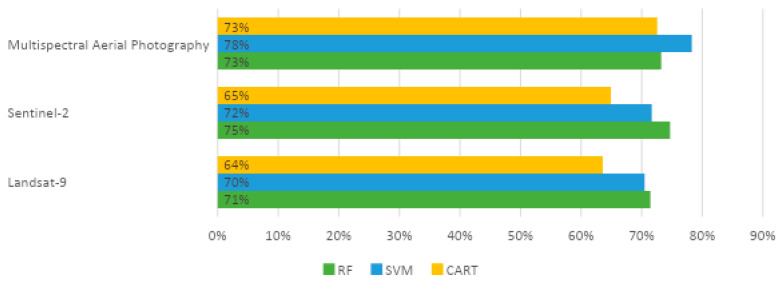
Overall accuracy of the tested machine learning algorithm.

The processing of benthic habitat data employs three machine learning techniques: RF, SVM, and CART. Figure 4 depicts the results of picture data accuracy testing with in situ data. The accuracy test results utilizing RF were the best of the three machine learning methods, as the 2022 Landsat 9 image, which has the highest RF accuracy of 71.43%, shows. The accuracy of the SVM is 70.48%. CART classification has the lowest accuracy of 63.55%. When using Sentinel-2 imagery in 2022, the accuracy findings are relatively comparable, indicating that RF has the highest level of accuracy, with an accuracy value of 73.68% and SVM of 71.76%. CART had the lowest accuracy value on Sentinel-2, with a rating of 64.91%. However, the most effective machine learning classification for multispectral aerial photography picture data was with an SVM of 78.28%. The accuracy results of RF and CART are not significantly different, 73.22% and 72.55%, respectively.

**Figure 5:**
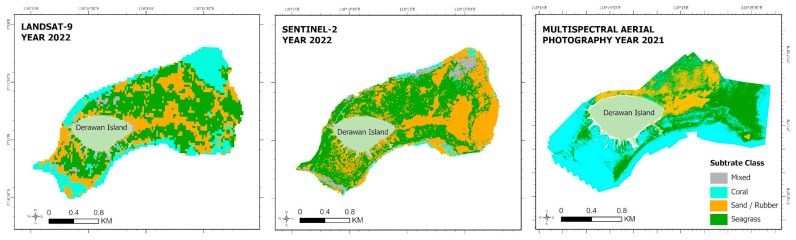
Comparison of the level of accuracy of RF classification with image data.

Figure 5 shows the distribution of the coral reef habitat using the RF algorithm over three tested images. The interpretations of object appearances in the Landsat 9 image data and Sentinel-2 image data are almost identical, and in one image capture area, on average, only 1 to 3 objects are identified, and differences or delineations between one object and another object can only be seen well at a low scale, so the detail of object interpretation is low. For instance, in the seagrass substrate, one object (sand) was identified in Landsat 9, and two objects (Seagrass and Sand) in Sentinel-2. This is due to the low spatial resolution of the Landsat 9 satellite image and the medium spatial resolution of the Sentinel-2 satellite image, which have spatial resolutions of 10 m and 30 m, respectively, where one pixel of image data represents a 10 × 10 or 30 × 30 m area.

The results of object interpretation using photos obtained by multispectral aerial photography are substantially more variable than the findings from the Landsat 9 and Sentinel-2 satellite data, as evidenced by one of the object interpretation data captures. By detecting more things and noticing differences between them, there can be a more accurate picture of what the objects in the field look like. That could be happening because the multispectral aerial photography’s ground sampling distance (GSD) is getting close to 8 cm/px. According to the results of the comparison of the object interpretations of the three image data sets above, there are considerable changes in spatial resolution that affect how well objects are classified using the resulting classification approach. High spatial resolution data are needed for data analysis needing fine detail since it can increase the degree of class variation and delineation of collected objects, resulting in the categorization and interpretation of exceedingly different data.

**Figure 6:**
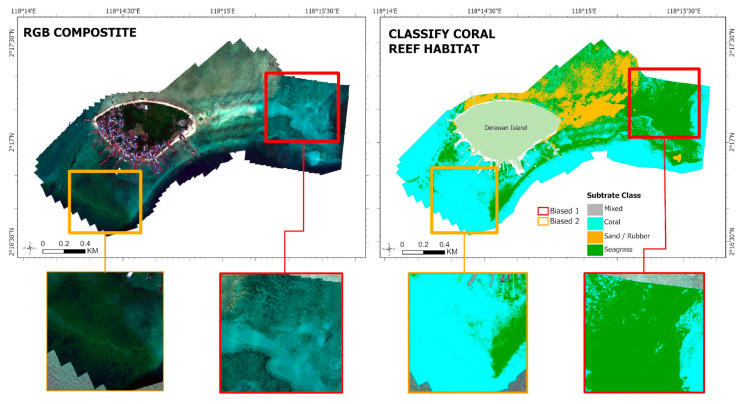
Comparison of Multispectral Aerial Photography: RGB composite and classification using RF algorithm.

The best machine learning classification results from Landsat 9 and Sentinel-2 image data appear more relevant and acceptable than the best machine learning classification results from multispectral aerial pictures using the SVM method. The result is due to significant bias in the classification findings obtained from multispectral aerial photography picture data, and field data validation reveals that the substrate categorization deviates significantly in some spots. Figure 6 depicts bias in the multispectral aerial photography picture data, with two examples of bias in the coastal area of Derawan Island.

Changes in weather or light during recording can cause bias in high spatial resolution imagery such as [51] Multispectral Aerial Photography photos. These could impact the recording outcomes, leading to biased or inaccurate data. The resolution difference between point data collecting methods like handheld GPS and multispectral aerial photography is another issue. The presence of ocean wave forces, which can lead to fluctuations in field sample placements, and the challenges of obtaining field sample data from seawater centers are the major causes of the resolution gap. Figure 6 explains the bias present in the map. Although it was recognized as a coral reef substrate in the red box area, the substrate in the field data is sand. Subsequently, the seagrass substrate is a coral reef substrate in the orange box. The biased portion of the box area thus leads to misclassification, which may lead to the reported events not being explained by the benthic habitat categorization created using multispectral aerial photography data. The RF classification algorithm may provide a higher level of accuracy when mapping benthic habitats than the SVM and CART algorithms, according to the classification results and accuracy attained. In this study, the benefit of the RF classification technique is its ability to produce a benthic habitat classification model with superior processing power over a large number of data dimensions. However, using RF on sample data with few dimensions would yield worse results; a preferable option would be to use the SVM classification technique.

In contrast to prior studies [15] employing WorldView-2 imagery to map benthic habitats using three machine learning methods (RF, SCM, CART), the classification in this research yielded 14 classes. Among the techniques utilized, RF demonstrated the most favorable overall outcomes, followed by classification tree analysis (CART), while SVM exhibited the least favorable results. The misclassification of certain areas occurred due to similar spectral characteristics and class descriptors. This phenomenon was observed in the current study as well, where classification results deviated from the actual conditions due to variations in color caused by weather conditions during image capture. It is known that the RF classification algorithm used on Sentinel-2 satellite images provides the level of accuracy in benthic habitat classification based on the accuracy test results given in Figure 4. With a validation accuracy value of 64.21% and a validation kappa value of 0.44, the accuracy test results demonstrate that the accuracy is satisfactory. The coral reef substrate generated in the accuracy validation has a producer accuracy of 52.63% and a user accuracy of 83.33%. In the accuracy validation, the accuracy level of the sand/rubble substrate formed was 75% with a producer’s accuracy and 54.55% with a user’s accuracy. Furthermore, accuracy validation was used to determine the accuracy of the seagrass substrate, with a producer’s accuracy value of 62.5% and a user’s accuracy value of 83.33%. Then the mixed substrate has a level of accuracy that is formed in accuracy validation with a producer’s accuracy value of 75% and a user’s accuracy of 21.43%

### 3.2. Temporal Pattern of Changes in Derawan Island Coral Reef Habitats in 2003, 2011, and 2021

Our analysis demonstrated that the RF classification algorithm was supremely accurate, primarily due to its capability to process large-scale data sets. In this study, we identified four distinct benthic habitat types: mixed, coral reef, seagrass, and sand or rubble. Utilizing this classification, we embarked on a detailed mapping of coral reef habitats. This was achieved by analyzing satellite data from three different years: Landsat 7 imagery from 2003 and 2011, and Sentinel-2 imagery from 2021. The insights gained from this mapping exercise laid the groundwork for an extensive examination of the spatial and temporal changes in Derawan Island’s coral reef habitats, with a particular focus on alterations in their size and composition over the years.

**Figure 7:**
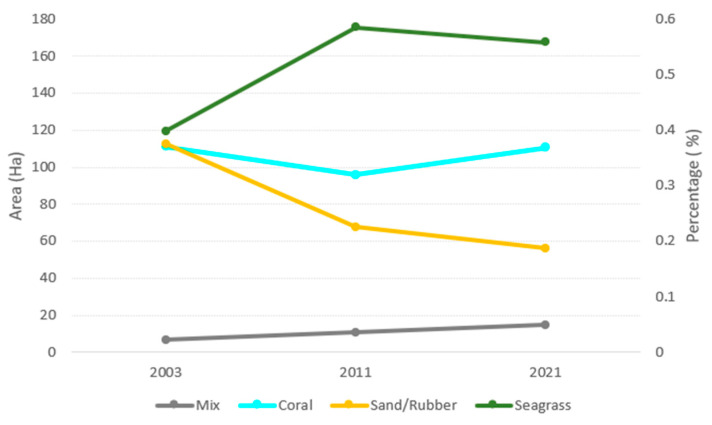
Percentage and Area Change in Derawan Coral Reef Ecosystem in 2003, 2011, and 2021.

The time series analysis of the benthic habitat on Derawan Island, illustrated in Figure 7, was derived from meticulous data processing. Our findings indicate a significant dominance of seagrass areas along the coastal region in 2003, occupying approximately 119.33 hectares and constituting approximately 34.13% of the island’s benthic habitat. The coral reef substrate, predominantly located around the island’s rim, covered an area of 111 hectares, accounting for 31.78%. Following closely, the sand/rubble areas spanned 112.5 hectares, making up 32.18% of the habitat. A detailed breakdown in Table S1 shows the mixed area being the smallest at 6.6 hectares, representing just 1.91%.

In 2011, the seagrass beds still dominated the coastal areas of Derawan Island, expanding to 175 hectares and comprising 50.20% of the habitat, as shown in Figure 7. The area covered by coral reef substrates experienced a decline of 15% to 20%, reducing to 95.8 hectares, primarily in the eastern section. Similarly, the sand/rubble habitats also saw a reduction, while the mixed habitat area increased slightly to 10.6 hectares, representing 3% of the total.

In 2021, as per the Sentinel-2 satellite imagery and subsequent classification resampled to a 30 m pixel size for compatibility with the Landsat 7 imagery, the seagrass beds continued to predominate. They expanded to 167.6 hectares, making up 48.02% of the habitat. There was also a noticeable increase in coral reef areas, particularly in the eastern region, totaling 110.5 hectares and forming 31.68% of the habitat. The sand/rubble and mixed habitats covered 56.1 hectares and 14.7 hectares, representing 13.24% and 4.40% of the area, respectively. This comprehensive analysis provides a nuanced understanding of the dynamic changes in Derawan Island’s benthic habitats over the observed periods.

**Figure 8:**
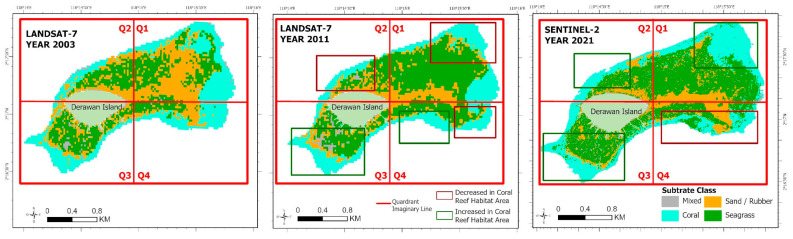
Changes in distribution of the benthic habitats of Derawan Island in 2003, 2011, 2021.

Similar to earlier investigations [23] that conducted analyses on temporal trends and spatial distribution, our study also performed analyses on both temporal and spatial distributions. However, our research uniquely divided the study area into four quadrants. Figure 8, titled ‘Pattern of Change in the Spatial Distribution of Benthic Habitats on Derawan Island from 2003 to 2021’, illustrates the analysis of changes in the benthic habitat distribution. An imaginary line in the form of a quadrant area, divided into four regions with distinct characteristics, was used for this analysis. Quadrants 1 and 4 are primarily influenced by natural factors, while quadrants 2 and 3 are more affected by human activities, with each quadrant covering an area of 200 hectares. This quadrant system aims to facilitate the analysis of changes in benthic habitat distribution on Derawan Island over the period from 2003 to 2021.

The analysis of Derawan Island’s benthic habitat classification highlights that the coral reef habitat is mainly concentrated in the reef crest zone, also referred to as the edge area. In this classification, the green polygons illustrate regions where the coral reef habitat has expanded, while the red polygons indicate areas of decline. From 2003 to 2011, quadrants 1 and 4, which are less developed and have minimal human activity, experienced significant deterioration in their coral reef habitats, totaling a loss of 15.3 hectares. In these less disturbed quadrants, the impact of natural forces, such as ocean currents and sea surface temperature changes, is more pronounced. The dominant current, moving from east to west, has been a crucial factor in altering sea surface temperatures over time, significantly influencing the coral reef habitats in these areas.

**Figure 9:**
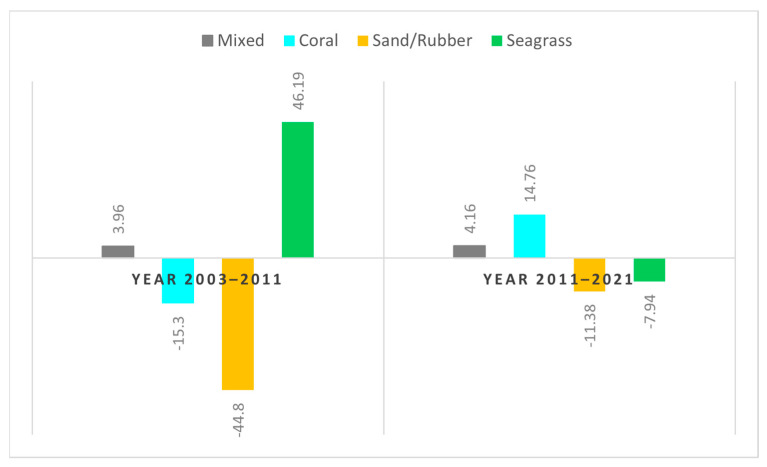
Changes in Ha of the distribution of benthic habitats on Derawan Island in 2003, 2011, and 2021.

Between 2003 and 2021, quadrants 2 and 3, situated closer to Derawan Island and more impacted by human activities due to higher population densities, experienced slight reductions in their coral reef habitat distributions along the reef slope. In contrast, from 2011 to 2021, there was a significant increase in the coral reef habitats in quadrants 1, 2, and 3. During this period, the coral reef habitats in these quadrants began to revert to a more natural state with reduced density, including in areas outside the barrier. This increase, predominantly observed in the reef crest or tuber area, contributed to the proliferation of diverse coral types, thereby enhancing the coral reef habitats around Derawan Island. The total expansion of the coral reef habitats in these quadrants from 2011 to 2021 was 14.76 hectares, aligning closely with the area measured in 2003. However, quadrant 4, an area with a lower population, continued to experience a decline in its coral reef habitat, influenced by natural factors such as ocean currents and changes in the sea surface temperature (Figure 9).

Statistical data on the temporal distribution of benthic habitats reveal a continuous reduction in the distribution of sand and rubble substrates. Conversely, from 2011 to 2021, the coral reef and mixed substrates experienced an increase in area, approximately 15 hectares and 4 hectares, respectively. Regarding the seagrass substrate, there was an initial increase from 2003 to 2011, followed by a decrease of approximately 8 hectares in the period from 2011 to 2021.

### 3.3. Coral Reef Density over a Decade on Derawan Island

**Figure 10:**
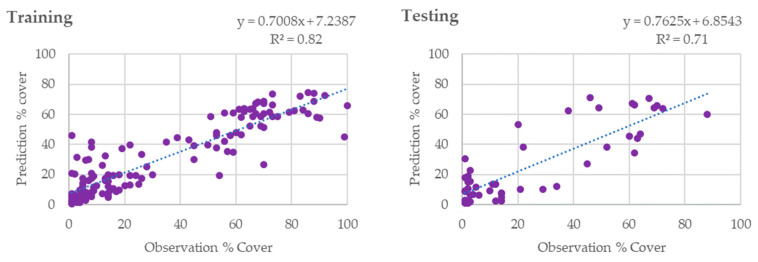
Creating the prediction model for coral density based on Sentinel-2 imagery 2021. Each dots represent the UPT data used to build the models.

**Figure 11:**
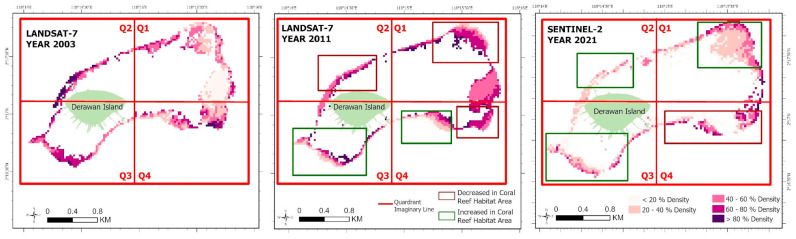
Changes in distribution of the coral density of Derawan Island in 2003, 2011, and 2021.

Figure 10 depicts the correlation between the observed and predicted percentage cover with a scatter plot, demonstrating a clear positive linear trend. The line of best fit for the training data is characterized by the equation y = 0.7008x + 7.2387, with an R-squared value of 0.8164, implying that the model successfully explains over 81% of the variance. This high level of variance explained indicates a robust predictive ability within the training context. Upon evaluation of the testing data, a similar positive linear relationship is evident, albeit with a modest reduction in the R-squared value to 0.7097. This best-fit line is represented by the equation y = 0.7625x + 6.8543. The observed reduction in the R-squared value, relative to the training phase, suggests a diminished, yet still substantial, predictive capacity when the model is applied to unseen data. Despite this diminution, the model maintains a commendable level of prediction accuracy, underscoring its utility in practical applications. Building on the established prediction model, Figure 11 extends the analysis to a temporal dimension, showcasing a time-series map of coral density across three distinct years: 2003, 2011, and 2021. This map provides a visual representation of the changes in coral density over the 18-year period, illustrating spatial patterns and trends within the benthic habitats of Derawan Island. In 2003, the map likely reveals a distribution of coral densities that corresponds to the initial baseline conditions within the study area. As we progress to 2011, the map reflects any shifts that have occurred over the eight-year interval, potentially showing changes in coral densities that may be attributable to various environmental factors or anthropogenic influences. By 2021, the culmination of the series, the map captures the latest state of coral densities, offering insights into the long-term evolution of the coral ecosystem. The changes depicted in the time-series map are underpinned by the predictive model’s accuracy, as previously discussed. The model’s capability to account for a significant portion of variance in the training and testing phases provides confidence in the reliability of the trends observed in the time-series map.

According to Figure 11, it is evident that the coral density on Derawan Island has undergone changes in various regions over an 18-year period. In 2011, there were fluctuations indicating both an increase and decrease in the extent and density of coral reef habitats. In comparison to 2003, quadrants 1, 2, and 4 experienced a decline in coral reef density in 2011. However, each quadrant exhibited distinct density changes, such as an average density increase from the 20–40% range to 60–80% in quadrant 1, and a decrease in average density from the >80% range to 40–60% in quadrant 2, as well as from the 20–40% range to <20% in quadrant 4. On the other hand, quadrants 3 and 4 witnessed an expansion in the coral reef habitat areas in 2011. In finer detail, the increased area was accompanied by an average increase from the 60–80% class to >80% in quadrant 3 and from the 20–40% class to 40–60% in quadrant 4. Overall, the period from 2003 to 2011 was characterized by a predominant reduction in the coral reef habitat area.

In contrast to the findings of the preceding year, the year 2021 is marked by a prevailing expansion in the spatial extent of the coral reef habitat. Over the ten-year period from 2011 to 2021, a discernible escalation in coral reef density is observed within quadrants 1, 2, and 3. Notable variations are particularly evident in quadrant 1, characterized by an extension of the elevated region towards the southwest, concomitant with an escalation in density from the 20–40% classification to the >80% classification. Furthermore, in quadrants 2 and 3, there is an enlargement in habitat area, albeit accompanied by a reduction in density from the 60–80% range to the 20–40% range and from the >80% range to the 20–40% range, respectively.

**Figure 12:**
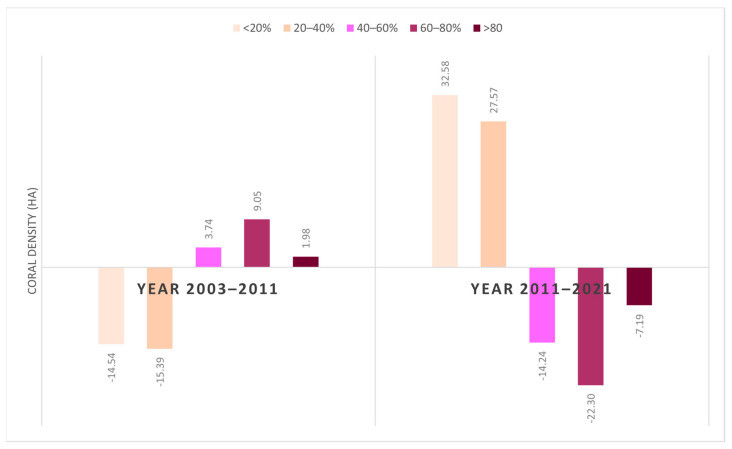
Changes in Ha of the distribution of coral density on Derawan Island in 2003, 2011, and 2021.

The overall analysis of coral density on Derawan Island reveals distinct trends between the periods 2003–2011 and 2011–2021 (Figure 12). From 2003 to 2011, there was a notable reduction in coral density within the <20% and 20–40% classes. Conversely, there was an expansion in the coral density area for classes exceeding 40%, with the most substantial increase observed in the 60–80% class. In contrast to the preceding period, the 2011–2021 timeframe demonstrates an inverse pattern. During this period, there was a significant growth in area for the 20% and 20–40% density classes. Simultaneously, a decrease in area was noted in density classes exceeding 40%, with the most substantial decline occurring in the 60–80% density class. In conclusion, the analysis of the coral density on Derawan Island indicates contrasting trends between the periods 2003–2011 and 2011–2021, marked by a decline in certain density classes and an increase in others, highlighting the dynamic nature of coral habitats over time.

## 4. Discussion

In our recent investigation into the benthic habitats of Derawan Island, we focused on the impact of spatial resolution on the accuracy of machine learning algorithms in classifying these habitats [52,53,54]. Utilizing three distinct methods—Random Forest (RF), Support Vector Machine (SVM), and Classification and Regression Trees (CARTs)—our study provided pivotal insights into their effectiveness with various types of image data [55,56,57,58,59,60]. Our results indicated that RF outperformed in terms of accuracy with Landsat 9 and Sentinel-2 imagery, while SVM showed notable efficacy in the context of multispectral aerial photography. This divergence in performance across different spatial resolutions and imaging types raises critical questions about the role of spatial resolution in classification accuracy and the potential biases inherent in remote sensing data [61], especially when faced with environmental variability, such as changes in light and weather conditions.

Our temporal analysis spanning the years 2003, 2011, and 2021, revealed significant shifts in the coral reef habitat types and densities [23,30,62]. These changes, shaped by both natural phenomena and human activities, highlight the dynamic and ever-changing nature of marine ecosystems [31,32,33,34]. The evolving landscape of coral reefs, as revealed through advanced imaging and sophisticated machine learning techniques, underscores the urgent need for adaptive conservation strategies that can respond to such dynamic changes.

The intricacies observed in our study extend beyond the mere technical aspects of image processing and machine learning. They touch upon a more profound understanding of the interplay between technology, environmental science, and conservation efforts. The varying degrees of classification accuracy across different algorithms depending on the image source signal the need for bespoke approaches in ecological studies. Our findings suggest that while RF is highly suitable for processing Landsat 9 and Sentinel-2 imagery, SVM excels in the realm of multispectral aerial photography, offering an avenue for detailed habitat analysis, albeit with a caveat regarding potential biases caused by environmental factors [61,63].

Moreover, our quadrant-based analysis of the coral reef changes over time provides an intricate perspective of the island’s complex ecosystem [44,62]. This approach revealed how different regions of the island are affected distinctly by natural forces and human activities, painting a comprehensive picture of the challenges and dynamics at play. Understanding these nuances is critical not only for comprehending the current state of Derawan’s coral reefs but also for informing future conservation strategies and management plans.

The study’s comprehensive approach not only deepens our understanding of the complex dynamics governing coral reef ecosystems but also brings to light the essential need for adaptive conservation measures. These measures are crucial for maintaining the ecological integrity of Derawan Island’s coral reefs in the face of persistent environmental challenges. By delving into both the image aspects and temporal distribution of coral reef cover and density, our analysis offers valuable insights into the fluctuating nature of this marine ecosystem. The observed changes over the years—likely influenced by a confluence of environmental conditions, human activities, and climatic factors—underscore the importance of ongoing monitoring and the identification of both resilient and vulnerable areas within coral reef ecosystems.

In conclusion, our study serves as a foundational step towards a more comprehensive understanding of marine ecosystem dynamics and the role of cutting-edge technology in environmental conservation. It highlights the importance of integrating advanced remote sensing and machine learning techniques with ecological expertise to develop more effective, responsive, and sustainable conservation strategies. The insights gained from Derawan Island’s coral reefs can be instrumental in guiding future research and conservation efforts, not only within this specific ecosystem but also in other marine environments facing similar challenges.

## 5. Conclusions

This study has made significant strides in understanding the spatial and temporal dynamics of coral reef environments on Derawan Island, leveraging a combination of satellite data, machine learning algorithms, and multispectral aerial photography. A standout finding of our research is the superior performance of the Random Forest (RF) classification method in analyzing these complex ecosystems. The RF algorithm demonstrated notable accuracy in classifying benthic habitats, distinctly outperforming other methods such as Support Vector Machine (SVM) and Classification and Regression Tree (CART) algorithms. Our analysis indicates that the accuracy of habitat classification is heavily influenced by both the spatial resolution and the quality of field data, satellite imagery, and multispectral aerial photographs. This underscores the importance of high-quality, high-resolution data in ecological studies.

In terms of spatial patterns, we observed a distinctive clustered formation of coral reefs along the edges of Derawan Island. This pattern provides insights into the habitat preferences and the distribution mechanisms of coral ecosystems. Temporally, our study reveals a concerning decline in both the area and density of coral reef habitats across all regions of the reef crest from 2003 to 2011. However, the period from 2011 to 2021 marked a turnaround, with an overall increase in the area of coral reef habitats throughout Derawan Island. This increase, however, is coupled with a general decline in coral density, an aspect that merits further investigation to understand the underlying causes and implications. The exception to this trend was observed in Quadrant 4, where different dynamics were noted. This variation highlights the complexity of coral reef ecosystems and the influence of various biotic and abiotic factors in shaping their growth and distribution.

In conclusion, this research not only sheds light on the changing landscapes of Derawan Island’s coral reefs but also establishes the efficacy of advanced technological approaches in ecological monitoring. The integration of RF classification with high-resolution spatial and temporal data has set a new standard in habitat analysis. Our findings call for continued and focused conservation efforts, particularly in areas showing a decline in coral density. Future research should aim to integrate additional environmental variables to further unravel the complexities of these ecosystems, ensuring the preservation and resilience of these vital marine habitats.

## Figures and Tables

**Table 1:** Data used for research.

Data	Resolution Time	Resolution Spatial	Time	Source
Landsat 7	16 Days	30 m	2003 and 2011	USGS
Landsat 9	16 Days	30 m	2022	USGS
Sentinel-2	5 Days	10 m	2021 and 2022	European Union/ESA/Copernicus
Multispectral Aerial Photography	One time	8 cm	2021	In-situ data
Underwater Photo Transects (UPT)	One time	-	2021	In-situ data

## Data Availability

The datasets are unavailable due to privacy and ethical restrictions.

## Supplementary Materials

The following supporting information can be downloaded at: https://www.mdpi.com/article/10.3390/s24020466/s1, Figure S1: Classification results of three different Machine Learning Algorithms over three types of imagery; Table S1: Confusion Matrix.
