# Supplementary material for: Assessing Derawan Island’s Coral Reefs over Two Decades: A Machine Learning Classification Perspective
Source: Sensors (Basel). 2024 Jan 12;24(2):466. doi: 10.3390/s24020466 (PMC10818429; doi:10.3390/s24020466)

Appendix

Appendix 1.

Table S1. Confusion Matrix.

Confusion Matrix Random Forest – Landsat 9

No	A	B	C	D	E	F	G
1	Object Class	Mixed	Coral	Sand / Rubble	Seag rass	Total	User's Accuracy
2	Mixed	3	1	0	2	6	50%
3	Coral	0	17	1	0	18	94,44%
4	Sand / Rubble	4	1	21	11	37	56,75%
5	Seagrass	4	0	9	39	52	75%
6	Total	11	19	31	52	113	69,05%
7	Producer's Accuracy	27%	89,47%	67,74%	75%		
	Overall Accuracy	70,79%					

Confusion Matrix Support Vector Machine – Landsat 9

No	A	B	C	D	E	F	G
1	Object Class	Mixed	Coral	Sand / Rubble	Seagrass	Total	User's Accuracy
2	Mixed	2	0	1	2	5	40%
3	Coral	1	12	1	1	15	80%
4	Sand / Rubble	2	1	14	18	35	40%
5	Seagrass	0	0	4	46	50	92%
6	Total	5	13	20	67	105	63%
7	Producer's Accuracy	40%	92,3%	70%	68,65%		
	Overall Accuracy	70,47%					

Confusion Matrix Classification and Regression Tree – Landsat 9

No	A	B	C	D	E	F	G
1	Object Class	Mixed	Coral	Sand / Rubble	Seagrass	Total	User's Accuracy
2	Mixed	0	2	2	1	5	0%
3	Coral	0	11	2	2	15	73,33%
4	Sand / Rubble	0	0	18	17	35	51,42%

5	Seagrass	0	0	11	39	50	78%
6	Total	0	13	33	59	105	50,69%
7	Producer's Accuracy	0%	84,61%	54,54%	66,1%		
	Overall Accuracy	64,76 %					

Confusion Matrix Random Forest – Sentinel-2

No	A	B	C	D	E	F	G
1	Object Class	Mixed	Coral	Sand / Rubble	Seagrass	Total	User's Accuracy
2	Mixed	0	0	1	2	3	0
3	Coral	1	1	0	1	3	33,33%
4	Sand / Rubble	0	0	15	6	21	71,42%
5	Seagrass	0	0	4	26	30	86,66%
6	Total	1	1	20	35	57	47,85%
7	Producer's Accuracy	0%	100%	75%	74,28%		
	Overall Accuracy	73,68%					

Confusion Matrix Support Vector Machine – Sentinel-2

No	A	B	C	D	E	F	G
1	Object Class	Mixed	Coral	Sand / Rubble	Seagrass	Total	User's Accuracy
2	Mixed	0	0	1	2	3	0%
3	Coral	2	1	0	0	3	33,33%
4	Sand / Rubble	0	0	13	8	21	61,9%
5	Seagrass	0	1	2	27	30	90%
6	Total	2	2	16	37	57	46,3%
7	Producer's Accuracy	0%	50%	81,25%	72,97%		
	Overall Accuracy	71,92 %					

Confusion Matrix Classification and Regression Tree – Sentinel-2

No	A	B	C	D	E	F	G
1	Object Class	Mixed	Coral	Sand / Rubble	Seagrass	Total	User's Accuracy
2	Mixed	0	0	0	3	3	0%
3	Coral	0	0	0	3	3	0%
4	Sand / Rubble	0	0	14	7	21	66,66%
5	Seagrass	0	0	4	26	30	86,66%
6	Total	0	0	18	39	57	38,33%
7	Producer's Accuracy	0%	0%	77,77%	66,66%		
	Overall Accuracy	70,17%					

Confusion Matrix Random Forest – Multispectral Aerial Photography

No	A	B	C	D	E	F	G
1	Object Class	Mixed	Coral	Sand / Rubble	Seagrass	Total	User's Accuracy
2	Mixed	0	0	0	0	0	0
3	Coral	0	458	0	6	464	98,7%
4	Sand / Rubble	0	12	236	3	251	94 %
5	Seagrass	0	250	5	236	491	48%
6	Total	0	720	241	245	1206	60,19%
7	Producer's Accuracy	0%	63,61%	97,92%	96,32%		
	Overall Accuracy	77,11%					

Confusion Matrix Support Vector Machine – Multispectral Aerial Photography

No	A	B	C	D	E	F	G
1	Object Class	Mixed	Coral	Sand / Rubble	Seagrass	Total	User's Accuracy
2	Mixed	0	0	0	0	0	0%
3	Coral	0	410	0	54	464	88,36%

4	Sand / Rubble	0	2	236	13	251	94%
5	Seagrass	0	128	4	359	491	73,11%
6	Total	0	540	240	426	120<br>6	45,8%
7	Producer's Accuracy	0	75,92%	98,33%	84,27%		
	Overall Accuracy	83,33<br>%					

Confusion Matrix Classification and Regression Tree – Multispectral Aerial Photography

N<br>o	A	B	C	D	E	F	G
1	Object Class	Mixed	Coral	Sand / Rubble	Seagras s	Total	User's Accuracy
2	Mixed	0	0	0	0	0	0
3	Coral	0	451	2	11	464	97,19%
4	Sand / Rubble	0	12	236	3	251	94,0%
5	Seagrass	0	244	5	242	491	49,28%
6	Total	0	707	243	256	1206	48,06%
7	Producer's Accuracy	0%	63,79%	97,11%	94,53%		
	Overall Accuracy	77,03%					

Confusion Matrix Sentinel-2 in 2021

N<br>o	A	B	B	D	E	F	G
1	Object Class	Mixe d	Coral	Sand / Rubble	Seagras s	Total	*User's Accuracy*
2	Mixed	0	0	1	8	9	0%
3	Coral	0	11	0	0	11	100%
4	Sand / Rubble	2	6	17	11	36	47%
5	Seagrass	0	0	2	37	39	95%
6	Total	2	17	20	56	95	38%

7	*Producer's Accuracy*	0%	65%	85%	66%		
8	*Overall Accuracy*						68,5%

Appendix 2.

Figure S1. Classification results of three different Machine Learning Algorithms over three types of imagery.

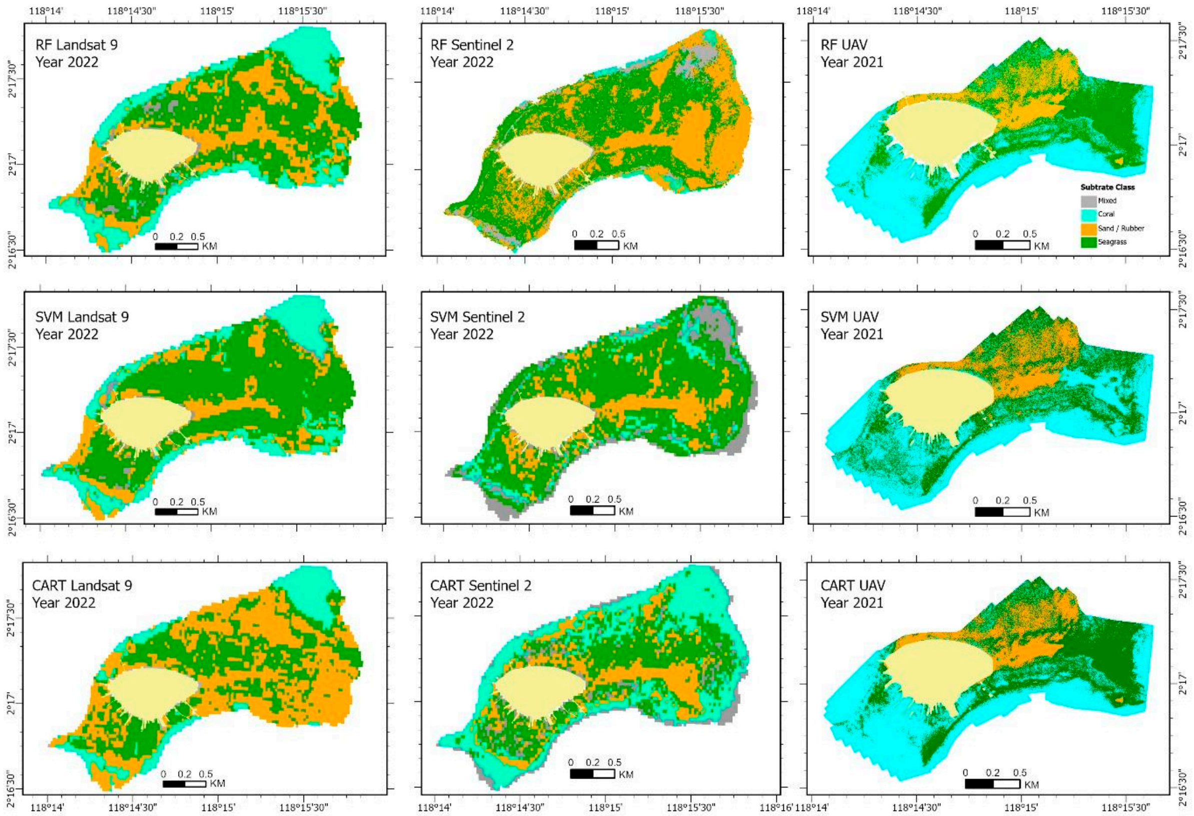

Supplement: Supplementary file 1 [file sensors-24-00466-s001.zip › sensors-2752086-supplementary.pdf]
